# Retraction: Grape Seed Proanthocyanidins Inhibit the Invasiveness of Human HNSCC Cells by Targeting EGFR and Reversing the Epithelial-To-Mesenchymal Transition

**DOI:** 10.1371/journal.pone.0210346

**Published:** 2018-12-31

**Authors:** The PLOS ONE Editors

Following publication, concerns were raised about similarities between this article [1] and an article published earlier by the same group in *BMC Complementary and Alternative Medicine* [2]. The two articles reported experiments addressing the same overall research questions but in different squamous cell carcinoma cell lines of tongue origin (OSC19 [1] and SCC13 [2]). The conclusions are largely overlapping, and there is substantial text overlap between the two articles in the Introduction, Discussion, and Conclusions. The *BMC Complementary and Alternative Medicine* article was not cited or discussed in the *PLOS ONE* article, and the two articles were under review during overlapping time periods.

In addition, concerns were raised about similarities between figures in the two articles:

- Fig 1A (FaDu panel) in the *PLOS ONE* article [1] is identical to Fig 1A (A431 panel) in [2], though these panels reportedly show results from different cell lines. The University of Alabama at Birmingham confirmed that there are no original data available to determine the cell line that was used in either article, and so the conclusion regarding the invasive potential of OSC19 cells versus squamous cell carcinoma cells from other head and neck sites is not supported.
- Fig 7 in the *PLOS ONE* article [1] is a modified version of Fig 6 in [2]. The *BMC Complementary and Alternative Medicine* article was not cited as the foundation of the *PLOS ONE* Figure.

Following a joint investigation by the Birmingham VA Medical Center and the University of Alabama at Birmingham, the institutions requested retraction of this article, as the conclusions could not be supported by available data. In light of the above issues, and in line with the institutions’ recommendation, the *PLOS ONE* Editors retract this article.

The authors did not comment on the retraction decision.
